# Pneumococcal carriage among HIV infected children in Accra, Ghana

**DOI:** 10.1186/s12879-017-2224-0

**Published:** 2017-02-08

**Authors:** Eric S. Donkor, Jennifer A. Annan, Ebenezer V. Badoe, Nicholas T. K. D. Dayie, Appiah-Korang Labi, Hans-Christian Slotved

**Affiliations:** 1 Department of Medical Microbiology, School of Biomedical and Allied Health Sciences University of Ghana, Accra, Ghana; 2 Department of Child Health, School of Medicine and Dentistry, University of Ghana, Accra, Ghana; 3 Department of Microbiology, Korle-Bu Teaching Hospital, Accra, Ghana; 4 Department of Microbiological Surveillance and Research, Statens Serum Institut, Copenhagen, Denmark

**Keywords:** Pneumococcus, Ghana, HIV, Carriage, Serotype

## Abstract

**Background:**

Pneumococcal carriage is the precursor for development of pneumococcal disease, and is also responsible for transmission of the organism from person-to-person. In Africa, little is known about the pneumococcus in relation to people with HIV infection. The aim of the study was to investigate the epidemiology of pneumococcal carriage among HIV infected children visiting a tertiary hospital in Ghana, including the carriage prevalence, risk factors and serotype distribution.

**Method:**

This was a cross sectional study carried out from February to May, 2015 at the HIV Paediatric Clinic of the Korle-Bu Teaching Hospital in Accra, Ghana. One hundred and eighteen HIV infected children were recruited and nasopharyngeal (NP) swabs were collected from them. Epidemiological data on demographic, household and clinical features of the study participants were also collected. The NP specimens were cultured for *Streptococcus pneumoniae* and the isolates were serotyped by latex agglutination. The data of the study was analysed using STATA 11 (Strata Corp, College Station, TX, USA).

**Results:**

Prevalence of pneumococcal carriage among the HIV infected children was 27.1% (95% CI: 19.1 to 35.1) and the only factor significantly associated with pneumococcal carriage was the presence of respiratory symptoms (OR, 2.63; CI, 1.06-6.53; *p* = 0.034). The most prevalent pneumococcal serotype among the study participants was serotype 19F (24.4%), followed by 16F (22%). Serotype coverage of the 13-valent Pneumococcal Conjugate Vaccine in this study was 41.5%. Multiple carriage of pneumococcal serotypes among the positive carriage cases was 34.3%.

**Conclusion:**

Pneumococcal carriage occurred in more than a quarter of the study population and was characterized by predominance of non-vaccine serotypes as well as a high prevalence of multiple carriage. Presence of respiratory symptoms appears to be a major determinant of pneumococcal carriage among the study population.

## Background

*Streptococcus pneumoniae* also referred to as pneumococcus, is part of the normal bacterial flora of the upper respiratory tract of humans. Carriage of the organism is affected by a wide range of factors such as age, acute respiratory tract infection and immunosuppression [1, 2]. An important characteristic of the pneumococcus is the presence of a polysaccharide capsule, which defines over 90 capsular types and is the basis of current pneumococcal vaccines [3, 4]. Clinically, the pneumococcus causes several invasive and non-invasive diseases including pneumonia, meningitis, septicaemia, sinusitis and acute otitis media. There are about one million new pneumococcal infections every year, majority of which occur in the developing world where children < 5 years are most affected, and the organism is responsible for 10–20% of all deaths in this age group [5]. HIV infected children have about forty times greater risk of invasive pneumococcal disease compared to healthy children; these infections are more likely to be fatal and importantly the incidence does not decline with age as in healthy people [6].

The vast burden of the pneumococcus underlies the importance of control through vaccination, and recently, pneumococcal conjugate vaccines are being introduced into the childhood vaccination programmes of many developing countries (http://www.gavi.org/, www.view-hub.org). Two types of pneumococcal conjugate vaccines (PCVs) are currently in use and they include the 10-valent vaccine which comprises pneumococcal serotypes 4, 6B, 9V, 14, 18C, 19 F, 23 F, 1, 5, 7F and the 13-valent vaccine which has three additional serotypes of 3, 6A, 19A [4]. Pneumococcal conjugate vaccines have been shown to be superior to the previous pneumococcal polyvalent polysaccharide vaccine (PPV 23) [7]. Though PPV23 contains 23 serotypes (1, 2, 3, 4, 5, 6B, 7F, 8, 9 N, 9V, 10, 11A, 12F, 14, 15B, 17F, 18C, 19A, 19F, 20, 22F, 23F, 33F), it provides limited protection in immunocompromised individuals and infants [7]. Despite current pneumococcal conjugate vaccines offering great hope in reducing pneumococcal disease burden, they are not a panacea for pneumococcal infections as the vaccines cover only a limited number of serotypes. It has been observed that non-vaccine serotypes of the pneumococcus have been increasing in prevalence since the introduction of the vaccines [8, 9], and therefore highlight the need for post vaccination surveillance.

Pneumococcal carriage is the precursor for development of pneumococcal disease, and is also responsible for transmission of the organism from person-to-person [10]. Recent pneumococcal vaccines are based on reducing pneumococcal carriage, hence incidence of pneumococcal disease [7]. Thus, pneumococcal carriage studies represent a suitable model for understanding host-pathogen interaction of the pneumococcus as well as providing vaccine related epidemiological data of this important human pathogen. In Ghana, the few studies carried out on pneumococcal carriage focused on either healthy populations [11] or the general population [12]. Consequently, there is hardly any data on pneumococcal carriage with regard to risk populations such as HIV patients. With the recent availability of routine pneumococcal vaccination in the country, there is an urgent need for the relevant epidemiological data on risk populations of pneumococcal disease, in order to inform vaccination policies. In this study, we present pneumococcal carriage data for HIV infected children attending a referral hospital in Ghana, thereby providing information on carriage prevalence, risk factors of carriage and serotype distribution.

## Methods

### Study site

This was a cross sectional study carried out from February to May, 2015 at the Paediatric HIV Clinic of Korle-Bu Teaching Hospital (KBTH) located in Accra, the capital city of Ghana. The population of Ghana is about 25 million people and the HIV prevalence is 1.6% [13, 14]. Korle-Bu Teaching Hospital is the largest hospital in Ghana, has eighteen departments and a bed capacity of 2000 [15]. The Child Health Department of the hospital has an outpatient attendance of 50–60 monthly, and the patients come from diverse socioeconomic backgrounds [15, 16]. The Paediatric HIV Clinic of KBTH, which is situated in the Child Health Department of the hospital, runs weekly with an attendance of about 40 patients [16]. Ghana initiated routine PCV13 immunisation in 2012 and the vaccine is given to infants at 6, 10, 14 weeks mainly through hospital based programmes [17, 18]. Currently, HIV infected children are not a priority for this vaccine in Ghana.

### Recruitment of study participants

Using a 95% confidence level, 81% estimated pneumococcal carriage prevalence reported previously [19], and 5% allowable error, 118 consecutive HIV positive children visiting the HIV clinic of KBTH were recruited in the study. Recruitment of the HIV positive children was based on the following inclusion criteria: informed consent from the primary caregiver, HIV-positive, and age < 15 years. Exclusion criteria were: presence of contraindications to a nasopharyngeal swab (such as thrombocytopenia); receipt of antibiotics in the last two weeks (except cotrimoxazole which pneumococci isolated in Ghana are completely resistant to [12, 20]).

### Specimen collection and interviews

Nasopharyngeal specimens were collected from the study subjects according to the World Health Organization guidelines by a qualified pediatrician [21]. The subject’s head was tilted slightly backward to straighten the passage from the front of the nose to the nasopharynx to make insertion of the swab easier. The swab passed directly backwards, parallel to the floor of the nasopharynx. The swab was passed through one nostril until it reached the posterior pharynx, which is approximately one-half to two-thirds the distance from the nostril to the ear lope. If resistance was encountered, the swab was removed, and the other nostril is tried, since the patient may have a deviated spectrum. The swab was allowed to sit in the place for 5–10 s and was rotated at 180° to saturate the tip before removing it slowly. The nasopharyngeal swabs were immediately immersed in 1 ml Skim Milk Tryptone Glucose-Glycerin (STGG) [21], and transported on ice to the laboratory within eight hours.

Using questionnaires, data were collected from the study participants on risk factors of pneumococcal carriage. The questionnaire covered three areas including demographic features (age, gender, nursery attendance) clinical features (recent respiratory symptoms, CD4 counts, antiretroviral treatment) and household characteristics (type of accommodation, number of household members, exposure to smoking).

### Characterization of *S. pneumoniae*

Briefly, the nasopharyngeal specimens were inoculated onto 5% blood agar plates, followed by an overnight incubation at 37 °C in 5% CO_2_ [21]. Suspected isolated colonies of pneumococci were Gram stained, and confirmed by bile solubility and optochin inhibition [11, 21–23]. Serotyping and detection of multiple serotypes was performed as described by Dayie et al. [11] The pneumococcal isolates were serotyped by the pneumotest latex agglutination kit (SSI Diagnostica, Hillerød, Denmark) and results confirmed by the Quellung reaction using serotype specific antisera (SSI Diagnostica).

### Data analysis

Data was analysed using STATA 11 (Strata Corp, College Station, TX, USA). Descriptive analyses including computation of arithmetic means, frequencies and percentages were done on the study variables. Univariate associations were performed between pneumococcal carriage and demographic, clinical and household features: analysis of variance was used for numeric variables, whereas chi-square test was used for categorical variables. Since only one variable emerged significant in the univariate analyses, multiple logistic regression was not carried out. Significance of variables was assessed by *p*-values, odds ratio and confidence intervals; *p* < 0.05 was regarded as significant. Serotype distribution was evaluated and impact of pneumococcal vaccination among the HIV children was estimated by the theoretical coverage of PCV 10, PCV 13 and PPV 23.

## Results

### Demographic, household and clinical features of the study participants

Demographic and household features of the hundred and eighteen (118) HIV positive children recruited in the study are reported in Table 1. The gender distributions of the study participants were similar and included 51.7% males and 48.3% females. Their mean age was 5.8 ± 3.3 years and majority of them were in the age group > 9–15 years (59.3%). Majority of them were Christians (83.9%), lived in compound houses (73.7%) and attended school (86.4%). The study participants lived in houses with an average of 17 people and 23% were exposed to passive smoking.

**Table 1:** Demographic and household characteristics of the study participants

Parameter	Number	%
Age (mean = 5.8 ± 3.3 years)		
< 5 years	21	17.8
5–9 years	27	22.9
≥ 9 years	70	59.3
Gender		
Male	61	51.7
Female	57	48.3
Current school attendance	102	86.4
Religion		
Christian	99	83.9
Moslem	19	16.1
Resident type		
Compound house	87	73.7
Self contained	31	26.3
No. of persons in house (mean = 17 ± 13)		
< 5	9	7.6
5–10	40	33.9
11–20	34	28.8
21–30	15	12.7
31–40	10	8.5
40	8	6.8
Exposure to passive smoking	23	19.5

As shown in Table 2, the mean CD4 counts of the study participants were 1088.9 cells/mm^3^ and 82.2% of them were on antiretroviral drugs. Only 11% had received the pneumococcal vaccine (PCV13). A proportion of 56.8% had respiratory symptoms and the most common symptom was cough (38.5%) followed by runny nose (25.6%). Otitis media occurred in 23.9% of the study participants while 4.3% were asthmatic.

**Table 2:** Clinical features of the study participants

Parameter	Number	%
Respiratory symptoms		
Difficulty in breathing	10	8.6
Cough	45	38.5
Runny nose	30	25.6
Sore throat	12	10.3
Asthma	5	4.3
Otitis media	28	23.9
Antiretrovirals taken	97	82.2
Pneumococcal vaccination (PCV13)	13	11

Mean CD4 counts of study participants = 1088.9 cells/mm3

PCV 13- Pneumococcal conjugate vaccine 13-valent

### Pneumococcal carriage and the associated factors

Overall 32 of the 118 study children carried pneumococcus, which translates to a carriage prevalence of 27.1% (95% CI: 19.1 to 35.1); carriage among the different age groups of < 5 years, 5–9 years and > 9–15 years were, 28.6% (95% CI: 20.5 to 36.8%), 51.9% (95% CI: 42.9 to 60.9%), 17.1% (95% CI: 10.3 to 23.9%) respectively.

In the univariate analysis, none of the demographic or household variables was significantly associated with pneumococcal carriage. The only clinical feature that was significantly associated with pneumococcal carriage was respiratory symptoms. In this case, individual respiratory symptoms including difficulty in breathing, cough, sore throat and cold did not affect pneumococcal carriage significantly. The overall combination of these respiratory symptoms was however significantly associated with pneumococcal carriage (OR, 2.63; CI, 1.06-6.53; *p* = 0.034). Previous receipt of pneumococcal vaccination or being on antiretroviral medication did not affect pneumococcal carriage significantly (Table 3).

**Table 3:** Univariate analysis of pneumococcal carriage with pneumococcal vaccination and antiretroviral treatment of HIV infected children

Parameter	Pneumococcal Carriage	OR (95% CI)	*p* value
Pneumococcal Vaccination			
Vaccinated	30.8% (4/13)	1.35 (0.39-4.75)	0.737
Unvaccinated	24.8% (26/105)		
Antiretroviral treatment			
On antiretroviral treatment	24.7% (24/97)	0.99 (0.29-3.35)	0.779
Not on antiretroviral treatment	19% (4/21)		

### Pneumococcal carriage serotypes

**Fig. 1:**
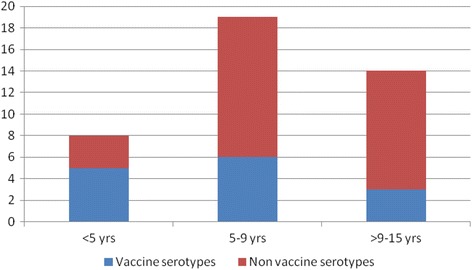
Distribution of vaccine and non-vaccine pneumococcus serotypes among different age groups of HIV infected children. Vaccine serotypes were relatively more common in the younger age group (< 5 years), while non-vaccine serotypes were more common in the older age groups (5–9 years; 9–15 years). However, there was no significant association between age and the distribution of vaccine and non-vaccine serotypes at *p* < 0.05

Serotyping was performed on 29 of the 32 pneumococci isolated from the study participants as 3 isolates had lost viability. Serotyping of the isolates yielded thirteen different pneumococcal serotypes and the predominant serotypes were 19F (24.4%) and 16F (22%) (Table 4). Serotype coverage of PCV10, PCV13 and PPV23 were 34.1, 41.5 and 46.3% respectively. The distribution of vaccine and non-vaccine serotypes of pneumococcus among different age groups of HIV infected children is illustrated in Fig. 1, which shows that there was no significant association between age and distribution of the two categories of serotypes. Thus vaccine and non-vaccine serotypes did not vary significantly among the different age groups including < 5 years, 5–9 years and > 9–15 years.

**Table 4:** *Streptococcus pneumoniae* serotypes isolated from HIV infected children

Serotype	Number	%	Serotype included in vaccine
6B	3	7.3	PCV-10, PCV-13, PPV-23
9V	1	2.4	PCV-10, PCV-13, PPV-23
19F	10	24.4	PCV-10, PCV-13, PPV-23
6A	3	7.3	PCV-13
10A	1	2.4	PPV-23
11A	1	2.4	PPV-23
15B	4	9.8	PPV-23
7C	2	4.9	Non-vaccine serotype
15C	3	7.3	Non-vaccine serotype
16F	9	22	Non-vaccine serotype
18B	1	2.4	Non-vaccine serotype
23A	1	2.4	Non-vaccine serotype
23B	2	4.9	Non-vaccine serotype

Total number of serotypes = 41; serotype data includes both single and multiple serotype carriage

Prevalence of carriage of multiple serotypes was 34.3% (11/32); all the cases of multiple serotype carriage involved two serotypes with the exception of one case where three serotypes were carried. In the multiple carriage events, 63.6% (7/11) co-carried at least one vaccine serotype and a non-vaccine serotype, 27.2% (3/11) co-carried at least one vaccine serotype and a non-vaccine serotype, and 9.1% (1/11) carried vaccine serotypes only.

## Discussion

In this study, the epidemiology of pneumococcal carriage among HIV positive children less than 15 years was investigated among outpatients at the Korle-Bu Teaching Hospital in Ghana. This study is to our knowledge the first report on pneumococcal carriage among HIV infected people in Ghana, and one of the few in sub-Saharan Africa. It is important to note that data from this study is post-vaccination data as the pneumococcal vaccine was introduced in Ghana in 2012. We observed an overall pneumococcal carriage prevalence of 27.1% whereas previous pre-vaccination data in Ghana reported carriage prevalence of 27–51% among children < 6 years [11, 12, 24, 25]. By comparison a study on HIV positive children in Tanzania reported a pneumococcal carriage prevalence of 80% among children < 12 years [19]. This Tanzanian study was carried out prior to the introduction of pneumococcal conjugate vaccines into the country in 2013, which may explain the high pneumococcal carriage compared to what this study found in Ghana. In Cambodia, Kremery *et al*. [26] reported a significant decline in pneumococcal carriage among HIV positive children following pneumococcal vaccination. Pneumococcal vaccination with PCVs targets younger children and this may explain the lower pneumococcus carriage prevalence among children < 5 years (28.6%) compared to those in the age range of 5–9 years (51.9%) in the current study.

The association of respiratory symptoms with pneumococcal carriage in this study has been previously reported for both HIV infected and healthy children [12, 19], and may be related to pneumococcal transmission events. Respiratory droplets transmit the pneumococcus either from healthy or sick people. In this study, the children with pneumococcal colonisation may have contracted respiratory symptoms from other people causing damage to the respiratory tract, which increases the chance of acquiring pneumococcus [10, 27]. This explanation is evident from a Tanzanian study that showed that pneumococcal carriage among HIV infected children was significantly associated with respiratory symptoms in their caregivers [19]. Interestingly, data from the current study shows that the relationship between pneumococcal carriage and respiratory symptoms is quantitative as a significant relationship between the two variables was observed only when the various respiratory symptoms were pooled. Further studies are required to throw more light on this, which could provide insights into pneumococcal transmission. Several risk factors of pneumococcal carriage, such as school attendance and history of acute asthma reported by other investigators [12, 27], were not observed in our study.

One of the predominant serotypes in the current study (19F) was also reported to be predominant among healthy children in Ghana during carriage surveys done before introduction of the pneumococcal conjugate vaccine in the country [11, 12]. However, the other predominant serotype (16F) in the current study has hardly been reported in Ghana. By comparison, pneumococcal serotype 19F was the most predominant serotype carried by HIV infected children in Kenya and Indonesia who had not received pneumococcal vaccination [28, 29]. Generally, the carriage serotype distribution in the current study is similar to that reported among healthy children in Ghana with exception of the serotype 16F predominance [11, 12]. In Kenya, a study showed that there was no significant difference in serotype distribution between HIV infected and uninfected children [28]. Serotype 16F is not covered by PCV13 and this largely accounts for the low serotype coverage (34.1%) of this vaccine in the current study. In Australian Aboriginal children, serotype 16F emerged as the predominant serotype in carriage and otitis media following implementation of PCV7 immunisation [30]. The evolutionary significance of serotype 16F among the HIV infected children in this study is difficult to explain as there is no pneumococcal prevaccination serotype data for HIV infected people in Ghana. It is likely that this serotype has emerged in evolution of the pneumococcus following introduction of PCV13. Whether this is truly the case would become evident in post-vaccination surveillance of pneumococcal serotypes involving healthy and at-risk populations in Ghana.

Prevalence of carriage of multiple serotypes in the current study (34.3%) seems very high compared to the 10% prevalence reported among healthy children in Ghana by Dayie et al. [11]. A study in Malawi showed that, prevalence of carriage of multiple serotypes among HIV infected children (44%) was higher than the prevalence among healthy children (34%) [31]. High prevalence of carriage of multiple pneumococcal serotypes enhances recombinational events between pneumococcus strains leading to evolutionary processes such as capsular switching which can result in vaccine evasion [32, 33]. Thus, the predominance of non-vaccine serotypes in multiple carriages observed among the study participants is of concern.

## Conclusions

This study presents to our knowledge the first post-vaccination data on pneumococcal carriage in Ghana. More than a quarter of the HIV infected children carried pneumococcus and the presence of respiratory symptoms appears to be an important determinant of pneumococcal carriage among the study population. There is a predominance of non-vaccine serotypes of the pneumococcus in carriage among the HIV infected children, particularly serotype 16F. Further studies are needed in the general population in Ghana to throw light on the evolution of pneumococcal serotypes following introduction of PCV13.

## Abbreviations

HIV: Human immunodeficiency virus
KBTH: Korle-Bu Teaching Hospital
NP: Nasopharyngeal
PCVs: Pneumococcal conjugate vaccines
STGG: Tryptone glucose-glycerin
